# M2e-Based Influenza Vaccines with Nucleoprotein: A Review

**DOI:** 10.3390/vaccines9070739

**Published:** 2021-07-04

**Authors:** Mei Peng Tan, Wen Siang Tan, Noorjahan Banu Mohamed Alitheen, Wei Boon Yap

**Affiliations:** 1 Department of Cell and Molecular Biology, Faculty of Biotechnology and Biomolecular Sciences, Universiti Putra Malaysia, Serdang 43400, Malaysia; meipeng27@gmail.com (M.P.T.); noorjahan@upm.edu.my (N.B.M.A.); 2 Center for Toxicology and Health Risk Studies, Faculty of Health Sciences, Universiti Kebangsaan Malaysia, Jalan Raja Muda Abdul Aziz, Kuala Lumpur 50300, Malaysia; 3 Department of Microbiology, Faculty of Biotechnology and Biomolecular Sciences, Universiti Putra Malaysia, Serdang 43400, Malaysia; wstan@upm.edu.my; 4 Laboratory of Vaccine and Biomolecules, Institute of Bioscience, Universiti Putra Malaysia, Serdang 43400, Malaysia; 5 Biomedical Science Program, Faculty of Health Sciences, Universiti Kebangsaan Malaysia, Jalan Raja Muda Abdul Aziz, Kuala Lumpur 50300, Malaysia

**Keywords:** influenza, matrix protein 2 ectodomain (M2e), nucleoprotein (NP), universal vaccine, adjuvant

## Abstract

Discovery of conserved antigens for universal influenza vaccines warrants solutions to a number of concerns pertinent to the currently licensed influenza vaccines, such as annual reformulation and mismatching with the circulating subtypes. The latter causes low vaccine efficacies, and hence leads to severe disease complications and high hospitalization rates among susceptible and immunocompromised individuals. A universal influenza vaccine ensures cross-protection against all influenza subtypes due to the presence of conserved epitopes that are found in the majority of, if not all, influenza types and subtypes, e.g., influenza matrix protein 2 ectodomain (M2e) and nucleoprotein (NP). Despite its relatively low immunogenicity, influenza M2e has been proven to induce humoral responses in human recipients. Influenza NP, on the other hand, promotes remarkable anti-influenza T-cell responses. Additionally, NP subunits are able to assemble into particles which can be further exploited as an adjuvant carrier for M2e peptide. Practically, the T-cell immunodominance of NP can be transferred to M2e when it is fused and expressed as a chimeric protein in heterologous hosts such as *Escherichia coli* without compromising the antigenicity. Given the ability of NP-M2e fusion protein in inducing cross-protective anti-influenza cell-mediated and humoral immunity, its potential as a universal influenza vaccine is therefore worth further exploration.

## 1. Introduction

Influenza A viruses (IAVs) are among the most medically important respiratory pathogens that infect various species of birds and mammals [1]. In spite of the availability of influenza vaccines, IAVs still cause high morbidity and mortality worldwide, especially during seasonal influenza outbreaks and influenza pandemics [2]. Every year, seasonal flu caused by IAVs results in approximately 3–5 million severe respiratory illnesses and 250,000–500,000 deaths globally [3].

Influenza viruses are categorized into four main types: A, B, C and D. They are enveloped viruses carrying 6–8 segments of single-stranded, negative-sense RNA genome and form the members of *Orthomyxoviridae* family [4]. Among influenza viruses, IAVs are the only type that can be further classified into subtypes based on the viral hemagglutinin (HA) and neuraminidase (NA) surface antigens [4]. So far, 18 HA subtypes and 11 NA subtypes have been identified. H1-H3 and N1-N2 subtypes have infected and circulated in the human population [5], with H1N1 and H3N2 currently being the co-circulating subtypes [6]. Likewise, influenza B (IBVs) and C viruses also infect humans, however they do not form multiple subtypes like IAVs; IBVs can be grouped into two antigenically distinct lineages, i.e., the Yamagata and Victoria lineages [4]. Both IBVs lineages have co-circulated in human populations since 1983 [7,8]. Since IAVs and IBVs are constantly present in human populations, they have been the main contributors to annual flu outbreaks. Influenza C virus usually causes mild flu in humans whereas influenza D virus is not known to cause human infections [9].

As proven in many infectious diseases, the influenza vaccination stimulates active acquired immunity against influenza infections, especially in individuals aged 6 months and older who do not experience contraindications [10]. However, the vaccine efficacies are often circumvented by the great mutational rates in influenza RNA genomes which then lead to the formation of new IAV strains through antigenic drift and antigenic shift [11]. Through these gene mutation events, the newly emerging IAV strains are able to escape from the pre-existing immunity induced by vaccination. Both antigenic drift and shift alter the antigenicity of the HA and NA glycoproteins without affecting their biological functions and protein conformation [2].

During antigenic drift, random mutations such as nucleotide substitutions, deletions and insertions that are introduced into the IAV RNA genome are selected via immune selection pressures and this results in antigenic changes in the virus surface proteins, i.e., HA and NA [11,12]. This dampens the prophylactic effect of the influenza vaccine as these two viral antigens constitute the major antigenic components in the vaccine formulation [13]. Antigenic shift, on the other hand, involves reassortment of genes across two or more different IAV subtypes infecting the same host; the gene reshuffling eventually results in a drastic change in the surface glycoproteins, hence altered antigenicity [14]. Worryingly, when antigenic shift occurs among IAV strains derived from different host species, for instance avian and human IAV strains, it may promote the formation of a brand new IAV subtype that potentially causes an influenza pandemic [15]. The novel influenza strain is antigenically different from the circulating influenza subtypes. It therefore reduces the efficacy of the currently used influenza vaccines [2]. Furthermore, novel influenza subtypes have been found to adapt well in humans, as observed in the most recent influenza pandemic in 2009 caused by a novel swine-origin H1N1 subtype [16]. Comparing young adults and elderly infected by H1N1pdm09, a higher mortality was observed in the former, including those who were otherwise healthy [17]. It is believed that elderly aged 65 and older might obtain some level of cross-protection from the pre-existing immunity triggered through previous exposure to H1N1 that had circulated in the past [18,19]. Nonetheless, pregnant women, children and individuals with chronic lung diseases, underlying illnesses and immunocompromised conditions are still at a higher risk of suffering from severe forms of infection outcomes, especially bacterial pneumonia [20,21]. In a nutshell, influenza vaccination is still the most effective preventive measure to circumvent seasonal and pandemic influenza and to avoid flu-related severe complications, especially in highly vulnerable individuals.

## 2. Influenza Pandemics

Until now, four severe influenza pandemics have been documented, which are the 1918 Spanish flu (H1N1), 1957 Asian flu (H2N2), 1968 Hong Kong flu (H3N2), and 2009 swine-origin flu (H1N1) [22]. The etiological agents of these pandemics signify a strong association of IAVs with flu pandemics, and hence are a major public health threat to global communities. Among the four reported flu pandemics, the 1918 influenza pandemic is deemed the worst infectious disease outbreak in history [23]. Many patients were reported to suffer from acute and adverse pulmonary hemorrhage and edema. The serious respiratory illnesses led to about 50 million deaths [24]. Some studies have endeavored to examine the genome of the 1918 pandemic IAV H1N1, but none were able to yield conclusive findings in regards to the host that served as the source of infection [25,26]. The genome of the 1918 IAV H1N1 is distinct from any avian or mammalian IAVs discovered so far whose RNA genomes are altered via random, silent nucleotide changes. The genome of 1918 H1N1 most likely evolved from an avian influenza virus through extensive gene adaptation in avian hosts prior to its spillover to human hosts [27].

Nearly four decades later, the second influenza pandemic struck the world populations in 1957. The pandemic was caused by a new IAV subtype (H2N2) that emerged through gene reassortment between human IAV H1N1 and avian IAV H2N2. The pandemic caused 1 million deaths [28]. About a decade later, in 1968, gene reassortment between human IAV H2N2 and avian H3 subtype resulted in a novel IAV subtype, namely H3N2 [29]. The novel IAV H3N2 is antigenically different from the previously identified pandemic IAVs. In this light, due to the lack of pre-existing immunity against the novel influenza subtype, the virus was significantly contagious and was able to spread rapidly and eventually led to a flu pandemic [30]. In the 1968 Hong Kong flu, approximately 1 million people died globally and among them, the elderly aged 65 years and older were the most affected population [29].

In 2009, the fourth flu pandemic caused by H1N1pdm09 resulted in 18,631 laboratory-confirmed deaths [31]. Genetic and epidemiological evidence indicated that the first pdmH1N1 outbreak occurring in Mexico was due to the pseudo-antigenic shift of H1N1 in which the divergence between H1 and H2 was proven to be 40–46% [32]. Owing to the genetic variation, the H1N1 strain evolved into a new influenza virus subtype (H1N1pdm09). The novel H1N1pdm09 virus was different from the circulating H1N1 strain and the seasonal flu vaccination therefore could not offer effective protection against the virus infection. The antigenic shift between a North American triple reassortant H1N2 and a Eurasian swine flu H1N1 gave rise to the novel H1N1pdm09 as a quadruple reassortant [33]. Among the eight viral RNA segments of H1N1pdm09, the *PB2*, *PB1*, *PA*, *NP*, *HA* and *NS* genes were closely related to that of H1N2. The North American H1N2 subtype was formed through two separate occasions of antigen shift in swine and was detected in 1999. It is a product of gene reassortment between a triple reassortant swine H3N2 (PB2, PB1, PA, NP, NA, M and NS) and a classical swine H1N1 (HA) [34]. The *NA* and *MP* gene segments of H1N1pdm09 were, on the other hand, derived from the avian-like Eurasian swine H1N1 [35]. According to genomic analysis findings by [36], the Eurasian swine H1N1 was also formed through at least two gene reassortment events, in which the *NA* and *MP* genes were derived from avian hosts in 1992 and 1997, respectively. When both of the reassortment products reshuffled their genetic materials in swine, a novel influenza A subtype finally emerged and was named H1N1pdm09.

## 3. Influenza Vaccination

Besides flu pandemics, seasonal flu outbreaks are reported worldwide each year. During these annual epidemics, the morbidity and mortality rates are highest among the elderly aged 65 years and older, children aged 2 years and younger, and individuals with underlying chronic medical conditions that place them at increased risk for influenza complications [37]. In this light, influenza vaccination is considered the most effective way to confer protection in all age groups against influenza virus infections [38]. At present, the commercially available influenza vaccine types include live attenuated, cold-adapted influenza vaccines (LAIV), inactivated influenza vaccines [39] and recombinant HA quadrivalent vaccine (e.g., Flublok Quadrivalent) [40] (Table 1).

LAIV encompass weakened influenza subtypes (IAV H1N1 and H3N2, IBV Yamagata and IBV Victoria) with very minimal risk to human health. The viruses are manipulated to adapt well at relatively cold temperatures (around 25 °C), and as a result, they only replicate poorly at human body temperature when injected intranasally into human subjects. It is generally accepted that LAIV are able to confer more prominent immune protection compared to inactivated vaccines. Owing to their ability to replicate poorly and temporarily in recipients, LAIV are effective activators of T-cell responses which in turn promote the activation of humoral and cell-mediated immunities (Table 1). However, even though those attenuated influenza viruses are harmless and safe for healthy individuals, they still may cause severe complications and side effects in immunocompromised people, especially those suffering from chronic medical conditions [41]. In this light, the administration of LAIV is recommended for those aged 2 years and older (Table 1).

In the formulation of inactivated influenza vaccines, the vaccines mostly contain detergent- or solvent-disrupted influenza viruses, whole inactivated virus, or purified HA and NA subunits [42] (Table 1). So far, the seasonal inactivated, quadrivalent vaccine is licensed for all persons aged 6 months and older without contraindications. Although the use of inactivated vaccines is recommended for a wider age range, their efficacies are usually relatively lower than that of LAIV. They stimulate mostly humoral (antibody) responses, hence confer lower immune protection against influenza virus infections (Table 1).

A recombinant influenza subunit vaccine known as Flublok Quadrivalent has been recommended by the Food and Drug Administration (FDA) for use in adults aged 18 years old and older since 2016 (Table 1). Its formulation consists of four HAs derived from the circulating H1N1 and H3N2, and two influenza B lineages [40]. The HA antigens are produced recombinantly using the Sf9 insect cell line. The amount of each HA antigen in Flublok Quadrivalent is three times higher than the standard inactivated influenza vaccine (45 μg vs. 15 μg). The higher amount of HA is believed to be the contributing factor to the cross-protective, longer lasting and improved immunogenicity against influenza virus infections [43]. Previous clinical trials proved that Flublok Quadrivalent was safe and did not result in adverse events in vaccinated individuals. The promising safety profile was supported by Woo and Moro [44] in which 95% of post-vaccination allergic and anaphylactic reactions were found to be non-serious. The predisposition to atopy was suggested to be the cause of the reported events. Therefore, it does not particularly imply that Flublok Quadrivalent is allergenic.

Several studies have shown that influenza vaccination can give protection to different populations including children [45,46,47], healthy adults [48] and the elderly [49,50,51]. In general, older adults or the elderly are recommended to receive high-dose trivalent inactivated influenza vaccine (TIV) (Sanofi Pasteur Fluzone^®^ (60 mcg ×3 strains)) or a standard dose of TIV (Fluad) with MF59 adjuvant [52]. MF59 is an oil-in-water emulsion of squalene oil which helps raise immune responses in recipients. In a nutshell, both of the high-dose and standard-dose adjuvanted vaccines are meant to boost protective immunity in older people, as approximately 90% of flu-related deaths involve the elderly, especially during influenza epidemics [53]. In several studies, elderly people aged ≥ 65 years old yielded lower post-vaccination antibody titers than healthy young adults after receiving inactivated, split-virus influenza vaccine [54,55,56]. On this note, the elderly are strongly encouraged to get vaccinated against influenza annually even though they may have been vaccinated earlier, as they are more susceptible to influenza infections than other populations [57]. A lower immune response against influenza vaccines could mean increased risks for severe complications and higher hospitalization rate and deaths due to influenza-related diseases such as pneumonia in older people, hence the importance of annual influenza vaccination.

Currently licensed influenza vaccines target the major surface antigens of influenza viruses, including the highly variable hemagglutinin (HA) and neuraminidase (NA). Given the error-prone nature of influenza RNA polymerase, one of the major challenges in the current influenza vaccine production is the unpredictable antigenic changes in HA and NA antigens [58]. Every year, the influenza vaccine formulation is revisited according to the upcoming dominant circulating IAV strains [59]. Consequently, it usually takes a long time for the production of influenza vaccine, in which at least a duration of 6 months is required for determining the dominant circulating influenza subtypes for the distribution of the vaccine for public use [60,61]. During this period, the initially predicted virus subtypes that were thought to dominate the next epidemic maybe undergo antigenic drift and introduce point mutations in their genomes. This may result in mismatching of the vaccine with the later circulating IAV subtypes. Eventually, the efficacy of the influenza vaccine declines as the circulating influenza subtypes fail to meet the prediction. This phenomenon was evident during an influenza outbreak in 2014–2015 in the United States. At that time, about 80% of the circulating H3N2 isolates were found antigenically and genetically dissimilar to the A/Texas/50/2012 (TX/12) strain used to formulate the 2014–15 North Hemisphere (NH) influenza vaccines. The vaccine effectiveness was estimated to be only around 20% [62]. Consequently, it led to high mortality and morbidity among people aged > 65 years old due to poor immunity against the virus infections.

In order to overcome the aforementioned challenges more effectively, newer vaccine candidates containing single or multiple antigenic components of influenza viruses that are capable of inducing wider, cross-reactive immunity against all or the majority of influenza viruses are highly needed [63]. More recently, influenza vaccine development has switched its focus towards the creation of universal influenza vaccine that can ideally confer protection against the majority of IAV strains, if not all, hence omitting the need for reformulation of influenza vaccines yearly. Amongst influenza antigens, the ectodomain of matrix protein 2 (M2e), which is conserved among all IAVs [64,65,66,67] has been extensively studied. The conserved M2e is part of the influenza surface antigens and it is scattered across the surface of the virus particles but shielded by the HA and NA antigens [68]. This characteristic renders M2e less exposed to influenza-specific antibodies, hence it is less prone to antigenic changes and is more conserved [68].

## 4. M2e as Immunogen

The influenza matrix protein 2 (M2) is an intramembrane viroporin which plays an important role during influenza virus replication. M2 is made up of 96 amino acids with an N-terminal ectodomain (M2e), a transmembrane domain and a cytoplasmic domain [69,70]. During IAV replication, the HA binds to host cell surface receptors, followed by endocytosis of influenza virions [71,72]. In the cytoplasm, the M2 protein is activated by the acidic endosomal environment and forms an ion channel that allows pumping of protons into the virion. Acidification of the interior of the virion loosens up the viral ribonucleoprotein (RNP) and its interaction with the matrix protein 1 (M1), and subsequently facilitates the migration of viral genome through the membrane fusion pores into the host cell cytoplasm where the viral RNA genome is transcribed and replicated [73,74,75].

**Figure 1:**
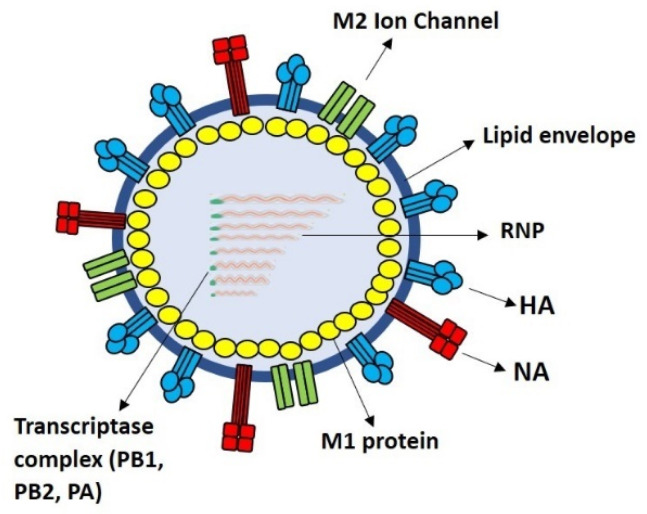
The M2 ion channels are present in small amounts on the surface of an influenza virion. They are shielded by neighboring HA and NA glycoproteins and hence are less prone to antigenic changes and are more conserved.

Being a conserved surface influenza antigen, M2e is a potential universal vaccine candidate for influenza. The interest in M2e is attributable to its relatively lower quantity and sequestered position, which in turn prevents it from antibody binding in comparison to the other larger and more abundant surface glycoproteins, i.e., HA and NA antigens (Figure 1) [76,77]. Generally, influenza HA to NA antigens are asymmetrically distributed on the viral surface with a ratio of HA:NA = 4–5:1, whereas only a few copies of M2e are present on the surface of an influenza virion [78].

In addition, the M2 protein is produced abundantly and presented readily on the surface of infected host cells [70,79,80]. In experimental models such as BALB/c mice and chickens, anti-M2e antibodies bind strongly to the surface-bound M2e, thereby inhibiting virus replication and budding from infected cells [79,81,82,83]. The binding of antibodies to M2e protein also promotes removal of infected lung epithelial cells via complement lysis or antibody-dependent cell cytotoxicity (ADCC) [84]. Previous studies proved that immunization with M2e antigen was able to provide cross-protection against lethal challenge with various IAV strains from the same host [83,85,86]. This is due to the relatively low mutation rate of M2e in which the vaccine-induced pre-existing immunity works effectively against homologous and heterologous influenza strains [87]. In a recent skin immunization strategy using a microneedle patch loaded with virus-like particles (VLPs) containing heterologous M2e domains, it was found to provide immune protection against human influenza H3N2; the lung viral load was reduced, and weight loss in laboratory animals was successfully prevented [88]. Although the M2e is well conserved among influenza viruses, the avian influenza viral M2e differs from that of human IAVs by several amino acids. This probably influences the specificity of M2e-based vaccines, thereby rendering partial or incomplete protection in IAV challenge studies [89,90]. To overcome the concern mentioned above, combining heterologous M2e domains in a vaccine design, for example, tagging two copies of each human and avian M2e with flagellin [Flagellin-2M2e (human)-2M2e (avian)] was found to protect BALB/c mice against lethal challenge with human IAVs (H1N1, H3N2) and avian influenza virus (H5N1) [87]. When introduced intranasally, the vaccine induced the production of mucosal IgA that binds to infected cells displaying the viral M2e on the cell surface, and subsequently inhibits the release of virions [91,92,93].

**Figure 2:**
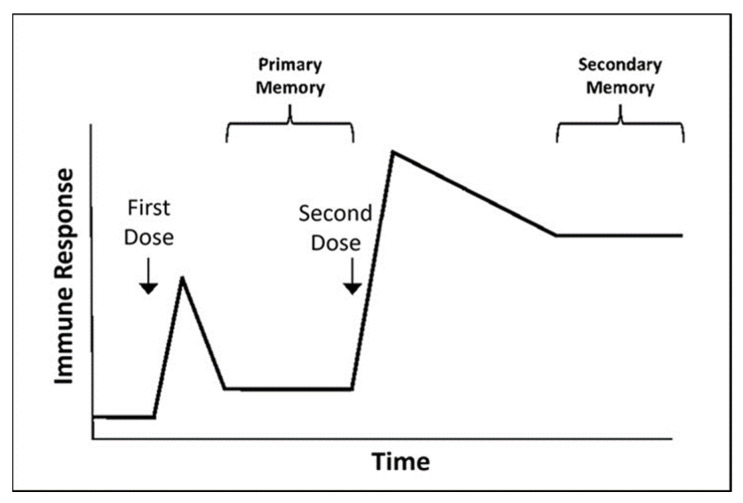
The prime-boost immunization strategy. The first dose of vaccine is given to activate the recipient’s immunity. In order to strengthen the recipient’s immunity, the second dose of vaccine is given approximately two weeks after the first dose. This strategy aims to stimulate previously activated memory immune cells to give heightened, long-lasting protective immunity.

In another study, primary infection of high-dose IAV H1N1 in pigs induced a very weak anti-M2e IgG response [94]. This observation implies the low immunogenicity of M2e and therefore explains why the first natural influenza infection normally induces relatively poor M2e-specific antibody responses. Six weeks later, the same pigs were reinfected with IAV H3N2. After the reinfection, the level of anti-M2e IgG was found to increase by over 10 folds. The heightened anti-M2e IgG level following the subsequent infection with a heterosubtypic IAV viral challenge supports the prime-boost immunization strategy that is aimed to boost human immunity against influenza viruses [83,95]. Basically, in a prime-boost immunization strategy, the first dose of vaccine is given followed by a second dose after two weeks (Figure 2). Immune cells (primary memory) activated through the first dose of vaccine are usually short-lived. In the boosting stage, the second dose of vaccine is given to boost the previously activated memory immune cells so that recipients develop long-lasting immune responses toward influenza viruses.

In order to enhance host immunity against influenza viruses using a M2e-based vaccine, Kim et al. [96] developed heterologous recombinant M2e5x VLPs. In the study, a tandem repeat of heterologous M2e peptides derived from human, swine and avian IAVs was constructed. Principally, the construction of M2e5x VLPs was intended to induce better protective M2e-specific immunity than a natural influenza virus infection. The greater immune protection was contributed to by the higher concentration of M2e antigens displayed on the VLPs than that of a natural virus unit. This eventually increases the recognition of M2e antigens on VLPs by the host humoral immune response [85,96]. In addition, the M2e5x VLP vaccination also directed the activation of both CD4+ and CD8+ T-cells that play essential roles in clearing the virus infection from the lungs and ensuring better recovery after influenza virus challenge.

Several clinical studies showed that M2e-based vaccines are safe and immunogenic in humans [65,97,98]. The humoral immune response triggered by M2e-based vaccines is responsible for anti-influenza effects [99]. This was evident in the passive transfer of anti-M2e monoclonal antibodies into animal models which in turn protected the animals against IAV challenge [100,101,102]. In addition, a study by Ramos et al. [98] further demonstrated that administration of a human monoclonal antibody directed against M2e in experimentally challenged human volunteers was able to reduce the influenza viral replication and clinical symptoms. Considering this evidence, it indicates that M2e protein is a good candidate for a universal influenza vaccine that could provide cross-protection against influenza virus challenges [83,85].

The influenza M2e peptide is known to have low immunogenicity in nature. It is often edited and manipulated in order to enhance the anti-M2e immune responses [89,103]. Examples include increasing peptide density (fusion of a number of M2e antigens in tandem form), fusing M2e peptide with carrier cargos such as hepatitis B virus core (HBc) antigen [104] and *Neisseria meningitidis* outer membrane protein complex (OMPC) [81] or co-administration with adjuvants such as flagellin [86] and cholera toxin [105]. Amongst those strategies, displaying M2e on a carrier has been acknowledged as an efficient, robust and cost-effective way to boost the immunogenicity of M2e [106]. It usually involves fusion of the coding sequence of M2e with that of the carrier, followed by recombinant protein synthesis and purification. At times, short linkers are added between the heterogeneous peptides to maintain their native structures or conformations [107,108,109]. The M2e peptide and carrier protein can also be synthesized and purified separately and subsequently linked together via a chemical ligand. In a previous study, a hepatitis B core (HBc)/M2e hybrid protein was expressed as an insoluble fraction in *Escherichia coli* (*E. coli*) [110]. In order to maintain the conformation of the hybrid protein, flexible glycine–serine-rich linkers were introduced at the junction points. This method successfully promoted the expression of soluble HBc/M2e chimeric protein in the bacterial cells [110].

A number of previous studies have demonstrated the fusion of M2e peptide to the HBc antigen via genetic manipulation. The fusion protein auto-assembles into VLPs displaying M2e peptides on their surfaces [104,111,112]. Besides HBc, a tandem repeat of M2e (M2e5x) was also fused to the transmembrane domain of influenza HA. The recombinant fusion protein assembled into VLPs and induced cross-protection against IAVs [113]. Surprisingly, the level of immune protection was even greater than that of HA vaccines [114]. The success of the transmembrane domain of influenza HA as an adjuvant carrier for M2e suggests the use of highly conserved IAV proteins that are able to assemble into particulate structure as the carrier cargo for M2e peptide [60]. It is believed that the immunodominance (T- and B-cell immunodominant epitopes) of the carrier protein can be transferred to M2e and increases the immunogenicity of the M2e peptide.

Among the IAV structural proteins, NP is believed to be a powerful carrier candidate for M2e. The influenza NP is a structural protein that encapsidates the viral negative-sense, single-stranded RNA genome. It is crucial for the viral RNA transcription, replication and packaging [73]. When synthesized in host cells, the NP subunits form two types of particles, i.e., the ring-like structures and long, filamentous complexes [115]. These robust conformations enable NP to display the M2e peptide steadily and enhance its immunogenicity.

## 5. Immunogenicity and Efficacy of Influenza Nucleoprotein (NP) as a Fusion Cargo for M2e

As an internal structural protein, the influenza NP has a relatively low mutation rate throughout the virus evolution [116,117]. The NP proteins of various virus isolates of the same influenza subtype have over 90% amino acid homology [118,119]. Comparing all four influenza types, the NP of IAVs and IBVs share a lower degree of homology. The major difference is found in the first 69 residues of IBV NP with no homology to that of IAVs; in addition, an extension of 50 conserved amino acids is present at the N-terminus of IBV NP but absent in that of the IAV NP [120].

During influenza virus infections, the NP is potentially recognized by cytotoxic T lymphocytes (CTL) [121,122]. The amino acid residues 55–69 are a T-helper (Th) epitope [123] while residues 147–158 are responsible for CTL response [124,125]. Both of the epitopes are somewhat restricted by the major histocompatibility complex (MHC) [126]; the higher the compatibility, the greater the immune response raised against the NP-based influenza vaccines [127].

**Figure 3:**
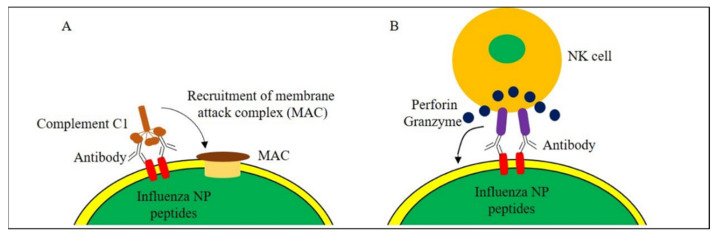
Complement- and antibody-mediated cytotoxicity in influenza virus-infected cells. (**A**) Binding of antibodies to NP peptides on the cell surface recruits complement factors to form membrane attack complex (MAC) on the cell membrane and induce cell lysis. (**B**) On the infected cell surface, NK cells bind to NP-specific antibodies and induce cell death via perforin and granzyme.

In infected cells, the NP is produced and later presented on MHC class I molecules on the surface of virus-infected cells. The NP-MHC-class-I complex interacts with the T-cell receptors (TcR) on CTL and thereby activates CTLs. The activated CTLs destroy influenza virus-infected cells via cell lysis and programmed cell death, hence elimination of the virus infection [128,129]. Due to the presence of conserved epitopes in the NP of influenza viruses, the CTL cross-reactivity can be directed against different influenza subtypes, and serves as a key factor to control influenza virus infections. The CTL cross-reactivity helps to inhibit virion release, control virus replication and reduce the severity of flu-related illnesses [130]. The Th-epitope, on the other hand, promotes the production of cross-reactive antibodies. The binding of cross-reactive antibodies to the NP disturbs the viral RNA transcription and genome packaging [131], eventually obstructing the viral infection processes. It is noteworthy that the cross-reactive antibodies also bind to the NP peptides displayed on the surface of infected cells via MHC molecules. The bound antibodies then trigger cell death via complement-dependent cell cytotoxicity (CDCC) (Figure 3A) or antibody-dependent cell cytotoxicity (ADCC) (Figure 3B) [63]. Recognition of membrane-anchored NP by antibodies recruits complement factors to form a membrane attack complex on the cell membrane and causes cell lysis (Figure 3A). In terms of ADCC, NK cells bind to NP-specific antibodies on the cell surface and induce cell death via effector molecules such as perforin and granzyme (Figure 3B). The presence of high titers of ADCC antibodies helps to reduce clinical symptoms and lower virus replication [132]. Besides CDCC, complement factors also enhance CD4+ and CD8+ T-cell responses, and maintain long-term memory towards influenza viruses [133,134].

The NP subunits assemble and oligomerize into particles with the amino end (N-terminus) facing outward from the NP particles [122,135]. Furthermore, it is widely proven that recombinantly produced VLPs do not carry genetic materials when produced in heterologous hosts, therefore they are less likely to induce severe allergic reactions and anaphylaxis [136,137]. The influenza NP has been produced recombinantly using different expression systems [138,139]. The recombinantly synthesized NP particles remain antigenic and immunogenic in experimental models. Altogether, this renders influenza NP an interesting antigen carrier for recombinant prophylactic vaccines against influenza viruses, especially those posing menacing pandemic threats such as H5N1, H9N2 or H7N7.

## 6. Expression of M2e-NP as a Fusion Protein in Heterologous Protein Expression Systems

Fusion of M2e with the NP protein can be achieved by (i) PCR gene fusion followed by the downstream production and purification of the recombinant fusion protein [107,108], or (ii) peptide synthesis technology that chemically links the M2e peptide to the NP. Nonetheless, bonding two heterologous proteins together by chemical linkages happens to deliver more foreign materials into vaccine recipients, and this may result in allergy in certain recipients [140,141]. Therefore, the production of antigenic subunits as vaccines via recombinant DNA technology has been on the rise in recent years [63]. For example, the construction and expression of nonstructural 1 (NS1) protein of IAVs on the surface of *Lactobacillus casei* strain C1 [142] and the display of the antigenic region of Nipah virus (NiV) NP on HBc VLPs [107]. In those studies, the AIV NS1 and NiV NP were linked to their respective carriers through PCR gene fusion and produced in large quantities in the *E. coli* expression system. Although produced in heterologous hosts, the viral antigens remained conformationally and antigenically intact.

Theoretically, displaying M2e peptides on NP particles can be achieved by fusing the M2e coding sequence to the 5′-end of the NP coding sequence. When expressed in *E. coli*, the fusion gene is translated into a chimeric protein in which the M2e peptide is present at the N-terminus of NP. The synthesized fusion M2e-NP protein is expected to oligomerize into NP particles displaying multiple copies of M2e on the particle exterior. Owing to its sheer advantages such as faster growth rate and higher yield with low-cost substrates [143], bacterial expression systems, e.g., *E. coli* expression system [144], are favored over the other alternatives such as insect [145], mammalian [146] and yeast expression systems [147] in many recombinant protein applications. Until recently, various recombinant proteins, peptides and vaccines have been produced in *E. coli* as it involves less tedious and laborious, cost-effective yet robust processes [148,149].

When multiple M2e-NP subunits assemble and oligomerize into particles, the overall number of M2e peptides present in the vaccine increases, which boosts the antigenicity and immunogenicity of M2e. Furthermore, the ability of NP to activate CD8+ T cells, Th and humoral responses is also transferred to the M2e. Altogether, the chimeric M2e-NP protein can stimulate greater humoral and cell-mediated responses, which in turn confer a broader, long-lasting protection against a wide spectrum of influenza strains [150]. The importance of vaccine-induced memory CD8+ T cells in minimizing influenza virus replication has been proven empirically [151]. Vaccine recipients manifested less severe symptoms with significantly improved clinical outcomes [152]. Similar observations describing the protective role of memory CD8+ T-cells were also documented in other animal models and human subjects challenged with influenza H1N1, H7N7, H3N2 and H5N1 viruses [150,153,154].

Besides the recombinant protein approach, delivery of M2e- and/or NP-based DNA vaccines using viral vectors is also described elsewhere [155]. The DNA vaccines induce protective immunity against influenza in recipients [156,157]. Delivery of NP and M2 DNA in conjunction with the adenovirus backbone protected mice challenged with a lethal virus dose [158]. It therefore bolsters the notion that protection induced by NP and M2 proteins concurrently is superior to that induced by a single protein or antigen [159,160]. Nonetheless, the use of DNA vaccines has been limited by its complex immunization processes and the production of DNA- and vector-specific antibodies [161]. The anti-vector antibodies may interfere with the immunization outcomes [162]. Moreover, the safety issue concerning chromosomal DNA mutation due to the integration of heterologous DNA molecules is still debatable [161]; as a result, the application of DNA-based vaccines requires rigorous empirical evidence to authenticate their safety profile and efficacy.

Owing to the safety concerns of DNA vaccines, another nucleic acid vaccine known as the mRNA vaccine can be attempted instead. Recently, mRNA vaccines have been used to prevent and generate herd immunity against SARS-coronavirus-2 (SARS-CoV-2) infections globally [163]. The use of mRNA vaccines for preventing the coronavirus disease (COVID-19) is based on the following advantages: (1) shorter manufacturing time, (2) relatively easier modification of the encoded antigen, (3) safety and (4) ability to stimulate both cellular and humoral immunity [164]. Considering those advantages, utilizing mRNA molecules that encode immunogenic antigens as potential vaccine candidates for preventing influenza could be a promising approach.

## 7. Potentials of M2e-NP Fusion Protein as a Universal Influenza Vaccine

Administration of recombinant M2e-NP fusion protein into subjects is expected to induce Th- [123,165] and CD8+ T-cell responses [125,158]. When the vaccinated subject encounters real influenza viruses, the pre-existing immunity is activated instantly by the viral M2e, and halts the virus replication in host cells. This subsequently prevents the spread of influenza virions to the vicinity. Furthermore, the M2e peptide and NP protein are well conserved among influenza virus types [60,64] due to relatively lower immune selection pressure. Given this benefit, the pre-existing immunity raised by M2e-NP fusion protein is able to cross-protect vaccine recipients against antigenically variable influenza viruses causing seasonal influenza and with pandemic potential. This strategy is an alternative to the currently licensed influenza vaccines that require annual reformulation in order to meet the next dominant circulating influenza subtypes [59], and therefore prevent antigenic mismatch, which is very likely to cause severe mortality and mobility rates.

In terms of protein production, the M2e-NP fusion protein can be produced in a heterologous expression host as a recombinant subunit vaccine; therefore, it does not contain live virus particles or viral genome that might pose health-threatening risks to immunocompromised individuals [43]. Furthermore, the manufacturing processes do not involve virus incubation in embryonated eggs hence avoidance of egg allergy issues. As a result, this type of vaccine is safe for all age groups. In addition, unlike chemically synthesized proteins or peptides with restricted structures and sizes, the antigenicity and immunogenicity of M2e-NP fusion protein is believed to have advantages such as (i) the NP protein is equipped with prominent T-cell epitopes, and (ii) subunits of M2e-NP protein are able to assemble into particles displaying multiple copies of M2e peptide exteriorly. Altogether, these properties make the M2e-NP fusion protein a potential candidate for universal influenza vaccines.

## 8. Conclusions

A universal influenza vaccine is urgently needed to give cross-protection against all IAV subtypes. Currently licensed influenza vaccines are facing a major concern: risk of antigenic mismatch that may reduce the efficacies of the vaccines. Furthermore, the vaccines only target circulating influenza subtypes without adequate protection against AIVs with pandemic potentials, such as avian influenza H5N1. In order to address the aforementioned concerns, a universal influenza vaccine must contain one or more conserved influenza proteins found in almost all influenza subtypes. The conserved proteins must be able to induce cross-protective cell-mediated and humoral immunity to minimize the severity of influenza infections. Both the influenza M2e peptide and NP protein fulfil the requirements for universal influenza vaccines. Combination of M2e and NP as a fusion protein is a promising formulation for a universal influenza vaccine that can provide broader and long-lasting protection against different influenza viruses.

## Figures and Tables

**Table 1:** Comparison between LAIV and inactivated influenza vaccines.

LAIV	Inactivated Influenza Vaccine	Recombinant HA Quadrivalent Vaccine (e.g., Flublok Quadrivalent)
Induces multifaceted immune responses including humoral (antibody) and T-cell responses.	Inclusive of viral major surface glycoproteins, i.e., hemagglutinin and neuraminidase. Induces mostly antibody responses.	Contains three times more HA than the standard inactivated influenza vaccine. It induces the production of anti-HA antibodies to prevent influenza virus infections.
Licensed for use in recipients aged 2 years and older.	Licensed for use in recipients aged 6 months and older.	Licensed for use in adults aged 18 years old and older.
Administered intranasally (mimicking natural infection).	Administered intramuscularly.	Administered intramuscularly.

## Data Availability

This study did not report any data.
